# Supplementary material for: Detection of m6A from direct RNA sequencing using a multiple instance learning framework
Source: Nat Methods. 2022 Nov 10;19(12):1590–8. doi: 10.1038/s41592-022-01666-1 (PMC9718678; doi:10.1038/s41592-022-01666-1)
Supplement: Supplementary file 1 — Supplementary Figs 1–5 and Supplementary Text. [file 41592_2022_1666_MOESM1_ESM.pdf]

nature methods

Article https://doi.org/10.1038/s41592-022-01666-1

Detection of m6A from direct RNA sequencing using a multiple instance learning framework

In the format provided by the authors and unedited

Supplementary Text

Comparison of *m6Anet* training procedures

As there are many more unmethylated sites than methylated sites as well as different relative methylation frequency for different kmer motifs across different datasets (Fig. 2c), we have trained several m6Anet models using different training strategies in order to assess the model generalisability over different cell lines and species. We perform this experiment on three different cell lines from two different species, ensuring that each model is validated on a set of genes not expressed in their respective training dataset. The sampling strategies we compare are as follow:

1. The m6Anet model ("**original**")
    a. In the original implementation, we oversample methylated positions to match the number of negative labels. This strategy maximises the amount of data used in m6Anet while maintaining the original relative k-mer frequency.
2. Undersampling of negative labels by kmer ("**undersampling**")
    a. This strategy ensures that the relative k-mer frequency is comparable between the positive and negative labels, at the cost of using less data (in particular for rarely modified k-mers many training data points will not be used)
3. Oversampling of positive labels by k-mers ("**oversampling**")
    a. This strategy ensures a comparable relative k-mer frequency between the positive and negative training labels. In contrast to the undersampling strategy, more data points are used.
4. Training on matched wild type and knockout data (using positive label positions only)("**matched knockout"**)
    a. In this strategy we train using only positions identified as modified, with positive labels using wild type cell line data, and negative labels from METTL3 knockout cell line data. This strategy ensures identical sequence context between positive and negative training labels, however it uses a minimum sequence context as none of the unmodified positions is used during training that make the majority of data points)

We applied these strategies to training models from the 2 human cell lines and the additional arabidopsis cell line, and we tested all models on all three data sets (Table 1). While the "oversampling" and "undersampling" strategies perform generally well, the "matched knockout" model performs poorly compared to the other models, most likely due to the limited sequence context the model is trained on compared to all

other models. The m6anet strategy as well as the “oversampling” strategy generalise well across species and data sets with very different k-mer profiles (Table 1). Overall these results suggest that the m6anet strategy is robust against a possible bias due to the methylated kmer frequency in the training data.

**HEK293T Test Set**								
**Training Cell Line**	**ROC AUC (original)**	**ROC AUC (Oversampling)**	**ROC AUC (Undersampling)**	**ROC AUC (matched knockout)**	**PR AUC (original)**	**PR AUC (Oversampling)**	**PR AUC (Undersampling)**	**PR AUC (matched knockout)**
HEK293T	0.828	0.774	0.664	0.547	0.343	0.271	0.151	0.084
HCT116	0.836	0.793	0.697	NA	0.349	0.280	0.174	NA
Arabidopsis VIRC	0.792	0.793	0.776	NA	0.311	0.314	0.281	NA
**HCT116 Test Set**								
**Training Cell Line**	**ROC AUC (original)**	**ROC AUC (Oversampling)**	**ROC AUC (Undersampling)**	**ROC AUC (matched knockout)**	**PR AUC (original)**	**PR AUC (Oversampling)**	**PR AUC (Undersampling)**	**PR AUC (matched knockout)**
HEK293T	0.903	0.859	0.775	0.582	0.466	0.327	0.213	0.079
HCT116	0.926	0.898	0.815	NA	0.498	0.380	0.278	NA
Arabidopsis VIRC	0.875	0.874	0.879	NA	0.383	0.389	0.387	NA
**Arabidopsis VIRC Test Set**								
**Training Cell Line**	**ROC AUC (original)**	**ROC AUC (Oversampling)**	**ROC AUC (Undersampling)**	**ROC AUC (matched knockout)**	**PR AUC (original)**	**PR AUC (Oversampling)**	**PR AUC (Undersampling)**	**PR AUC (matched knockout)**
HEK293T	0.881	0.877	0.896	0.674	0.267	0.249	0.284	0.049
HCT116	0.886	0.898	0.897	NA	0.237	0.238	0.227	NA
Arabidopsis VIRC	0.940	0.937	0.933	NA	0.389	0.346	0.284	NA

Table 1 Comparison of mAnet-based models trained on different cell lines with different sampling strategy on the HEK293T Test Set

Threshold Recommendation for selecting m6A sites and m6A reads

In order to achieve results with high precision, we recommend a threshold of 0.9 for selecting modified m6A sites as this achieves a good level of precision based on m6ACE-seq[1], miCLIP[2], as well as sensitivity to METTL3-KO data predicted by xPore[3]. To obtain more sensitive results, a lower threshold can be selected that returns a higher number of m6A sites at the expense of lower model precision, which might be beneficial, depending on the specific scenario in which m6Anet is applied to.

To detect m6A stoichiometry, we recommend a threshold of 0.0333 for determining whether a given read is modified. This threshold is selected based on m6Anet single molecule prediction results on the curlcake datasets[4] but can be changed by the users (https://m6anet.readthedocs.io/en/latest/)

Supplementary Figures

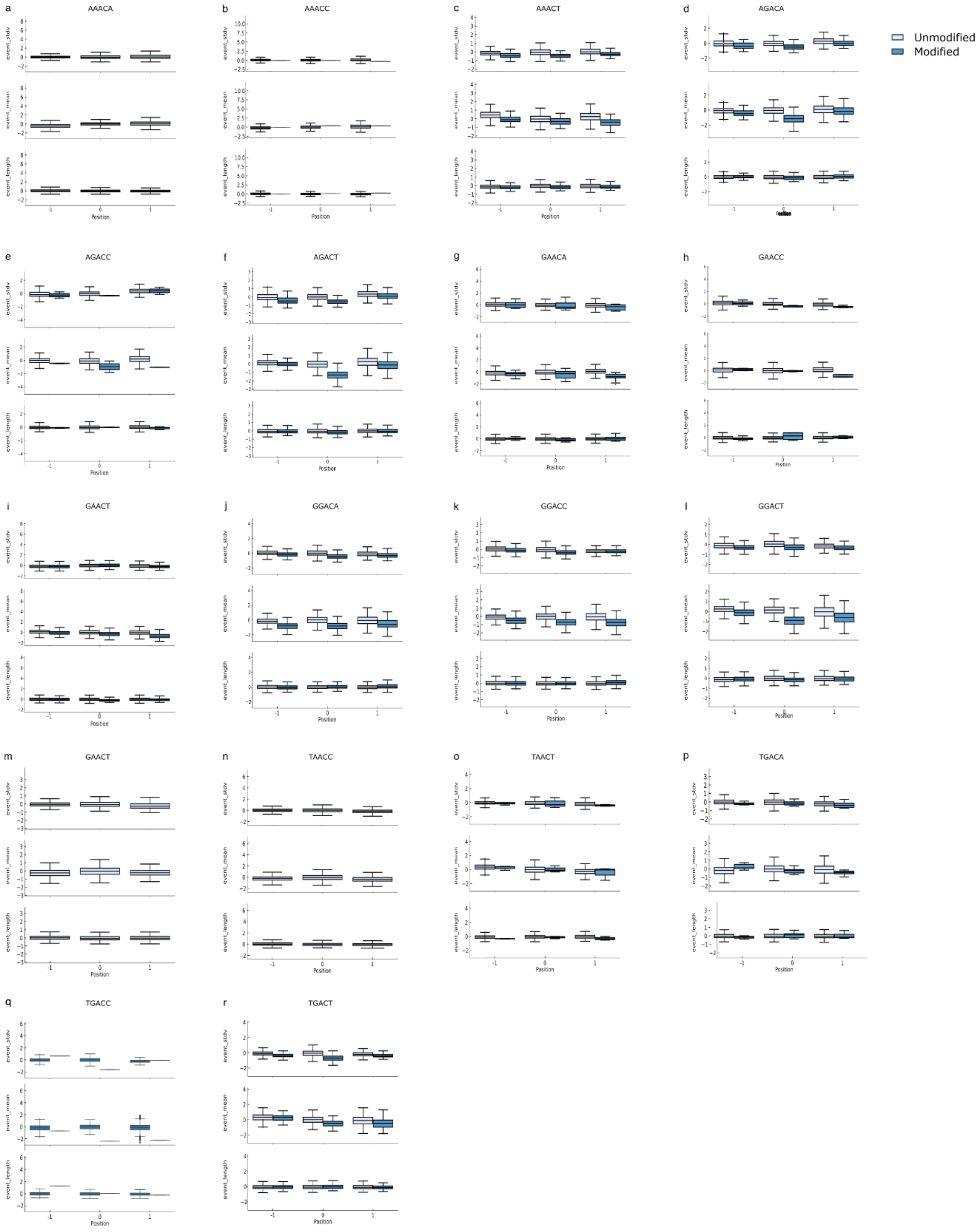

Supplementary Figure 1. (a-r) Box plot showing the difference in average features distribution between different *m6Anet* prediction across all 5-mers with with n=15,921. The horizontal lines on the boxes show minima, 25th percentile, median, 75th percentile, and maxima. Points that do not fall within 1.5 times of the interquartile range are considered outliers and are not shown on the plot

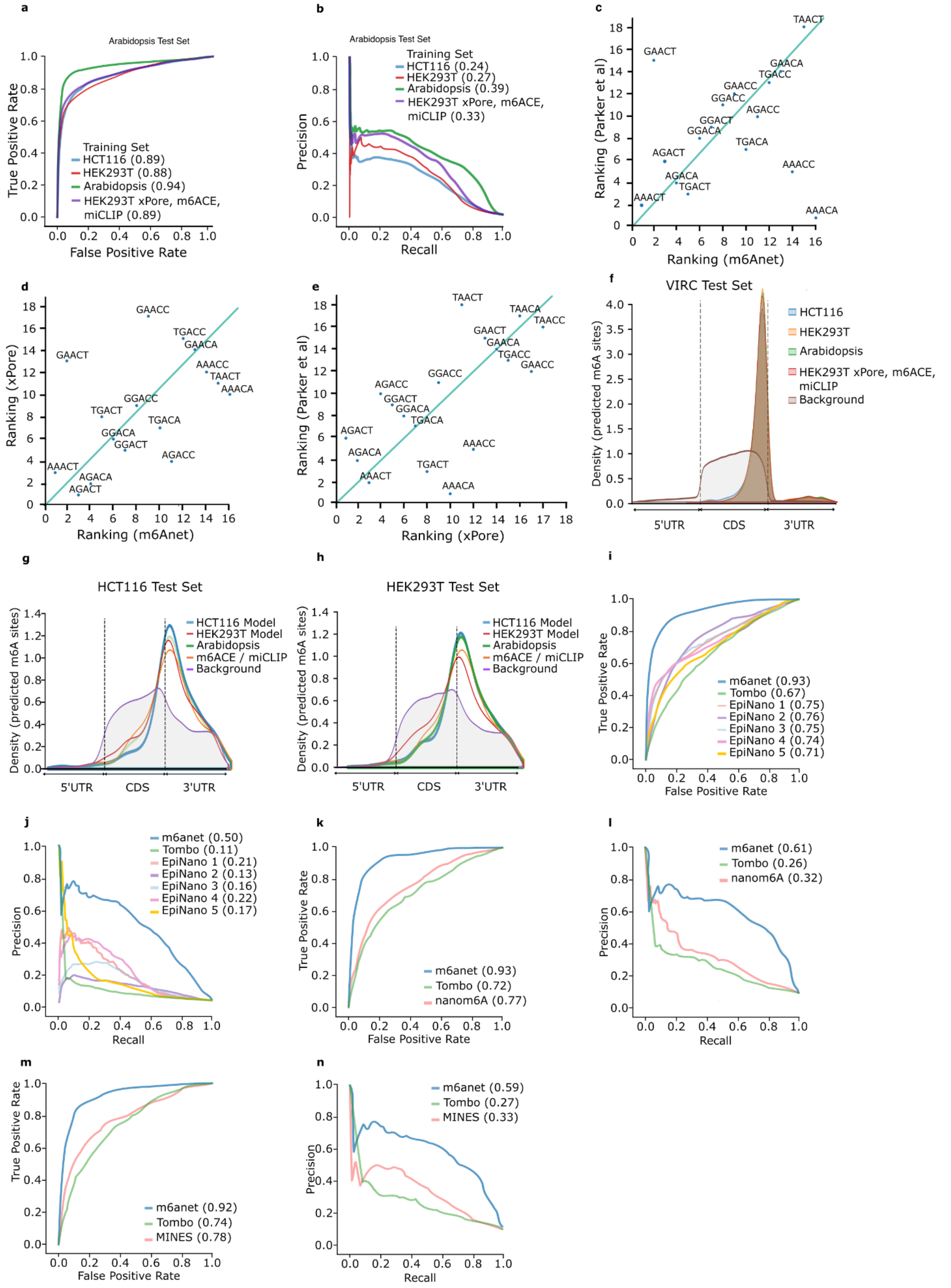

Supplementary Figure 2. Performance comparison between *m6Anet*, m6ACE-seq and miCLIP on HEK293T cell line. (a-b) ROC Curve and PR Curve of four m6Anet models

trained on HCT116 cell line, HEK293T cell line, Arabidopsis VIR-1 complemented cell line, and HEK293T cell line with the inclusion of KO sensitive positions as detected by xPore on the Arabidopsis VIR-1 complemented cell line test set. (c) Scatter plot comparing the frequency ranking of predicted motifs by m6Anet against Parker et al and (d) xPore and (e) xPore against Parker et al. (f-h) Metagene plot of m6Anet predicted sites on (f) VIR-1 complemented cell line (g) HCT116 and (h) HEK293T. (i-j) ROC Curve and PR Curve of *m6anet* against all 5 EpiNano models and Tombo. (k-l) ROC Curve and PR Curve of *m6anet* against *nanom6A* and *Tombo*. (m-n) ROC Curve and PR Curve of *m6anet* against *MINES* and *Tombo* on the HCT116 test set

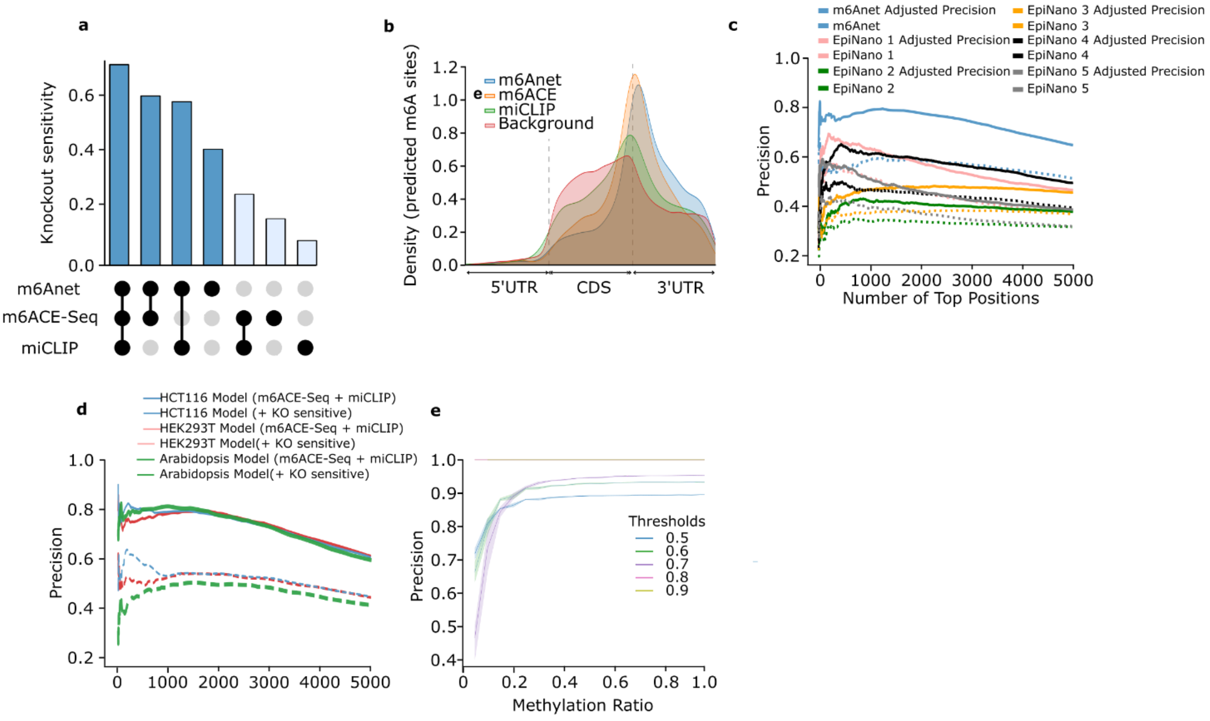

Supplementary Figure 3. Performance comparison between *m6Anet*, m6ACE-seq and miCLIP on HEK293T cell line. (a) Percentage of captured sites that show significant shift in signal distribution against METTL3-KO for each of the three protocols defined as sites with p-value < 0.05 as quantified by a two-tailed Welch's t-test between wild-type samples and METTL3-KO samples after multiple testing correction using Benjamini-Hochberg procedure. (b) Metagene plot of the modified sites captured exactly by one of the three protocols against the background distribution of all DRACH sites in the data that has at least 20 reads. (c) The adjusted precision after including position sensitive to METTL3-KO of *m6Anet* and all 5 EpiNano models and (d) m6Anet trained on HCT116, HEK293T and Arabidopsis VIRC cell lines. METTL3-KO sensitive sites are defined as those sites displaying statistically significant difference (p-value < 0.05) in current level between wild-type samples and KO samples after multiple testing correction using Benjamini-Hochberg procedure and p-value is quantified using xPore. (e) Precision of m6Anet on curlcake dataset under different threshold values for the site level probability predicted by m6Anet.

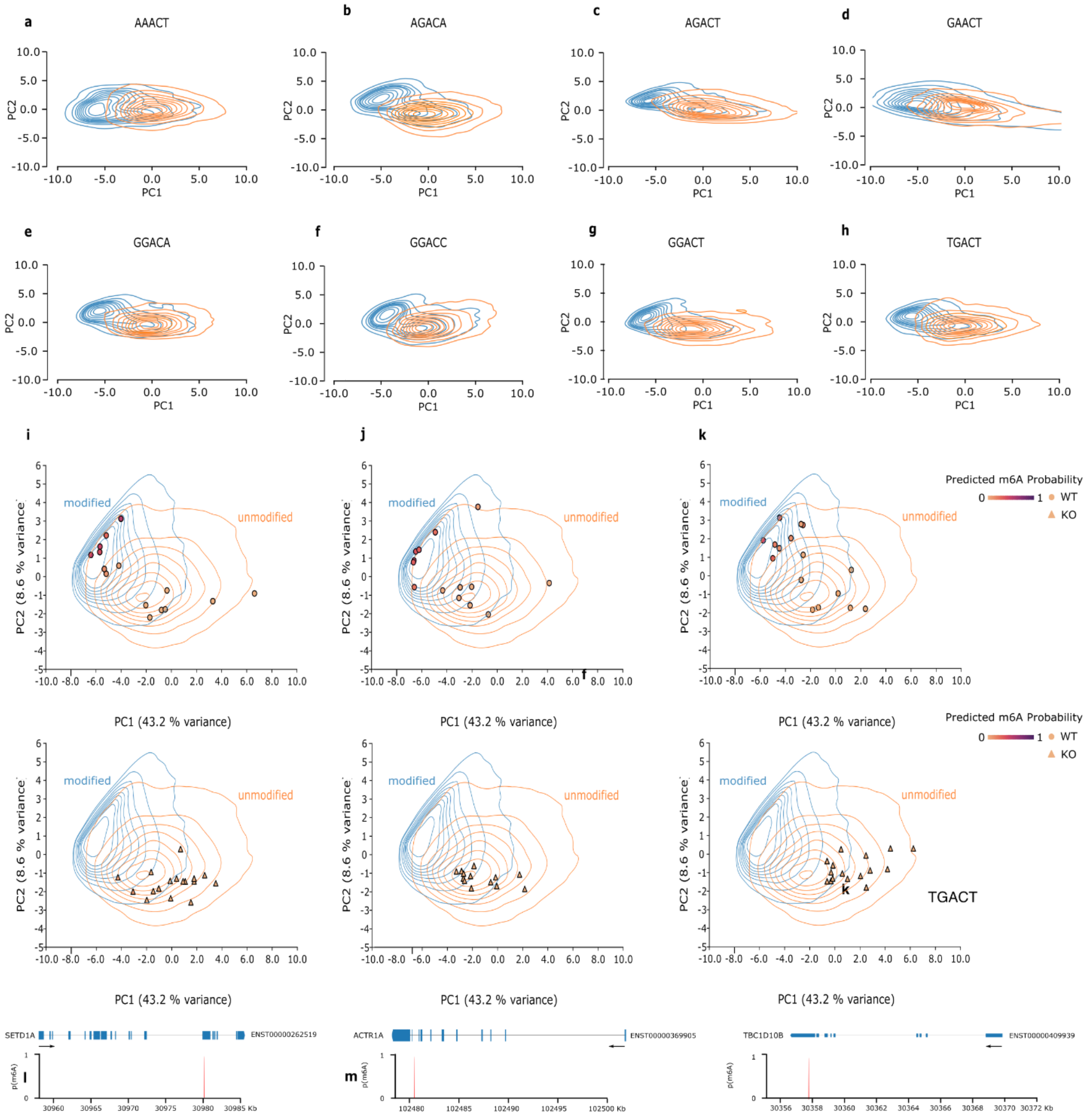
a
AAACT
b
AGACA
c
AGACT
d
GAACT
e
GGACA
f
GGACC
g
GGACT
h
TGACT
PC1
PC2
i
j
k
modified
unmodified
Predicted m6A Probability
WT
KO
PC1 (43.2 % variance)
PC2 (8.6 % variance)
TGACT
SETD1A
ENST00000262519
ACTR1A
ENST00000369905
TBC1D10B
ENST00000409939
l
m
p(m6A)
Kb

Supplementary Figure 4. Quantification of m6Anet on HEK293T cell line

Experimental design: We sample 100 reads from each DRACH sites and extract the output from the second last layer of *m6Anet* for each of these reads and visualize them on two dimensional space using PCA (a-h) Density plots of selected DRACH 5-mers that contain at least 20 modified sites (m6anet probability score $p >= 0.9$ on WT samples) and at least 20 unmodified sites (m6anet probability score $p <= 0.2$ on KO samples). Blue coloured density plots represent the wild type clusters while orange coloured density plots represent the knockout clusters. (i-k) Scatter plot of randomly sampled reads on the second, third, and fourth ranked positions from the wild-type (top) and METTL3-KO (bottom) cell lines, sorted by predicted modification probability on the wild type sample after the filter along with the corresponding density plot from curlcake datasets.

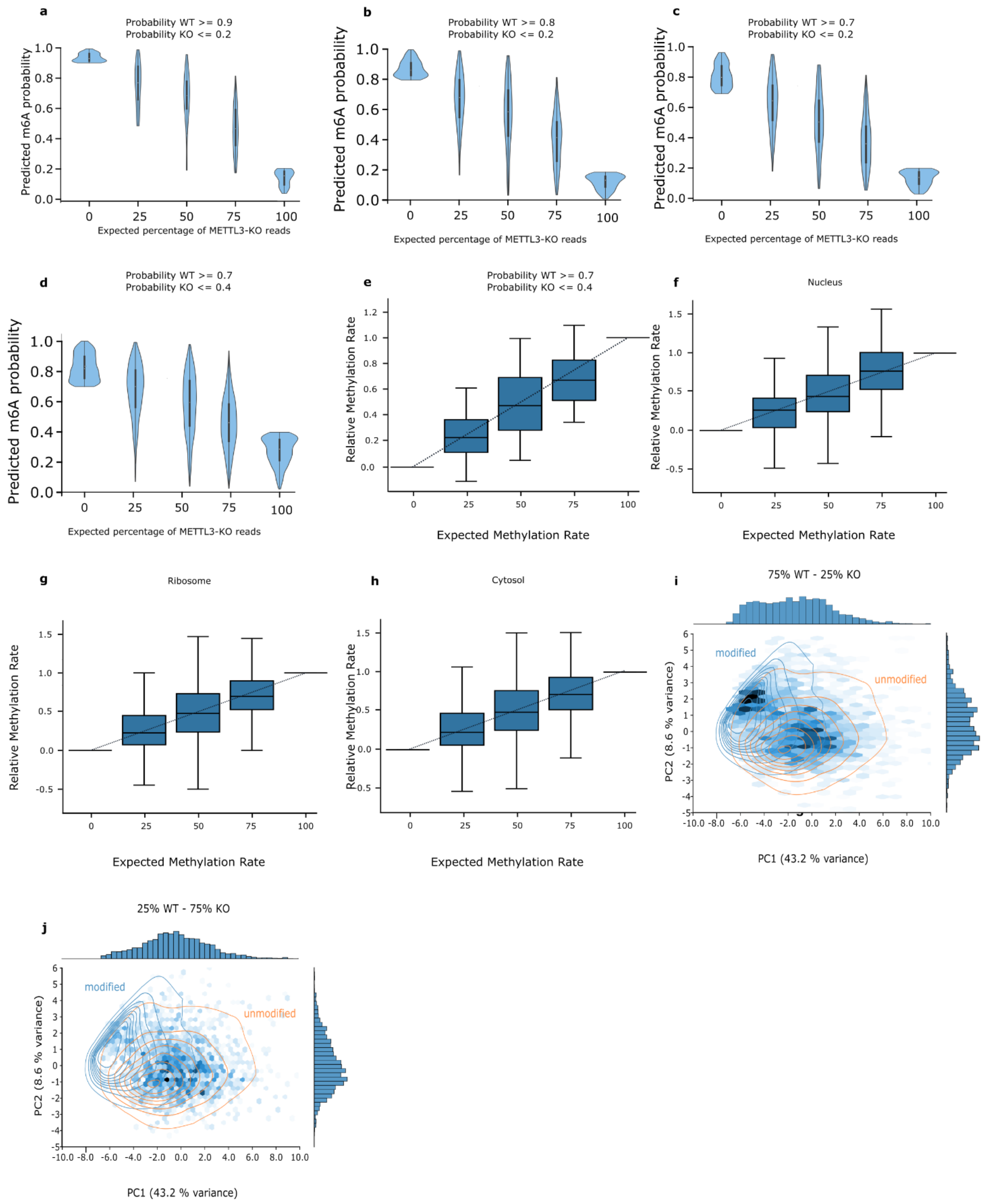

Supplementary Figure 5. Changes in expected representation and predicted probability on the HEK293T Knockout Mixtures

Experimental design: We extract 100 reads from positions that show both high probability of modification on the Wild Type sample and low probability of modification on the corresponding KO sample and expressed across all 5 Wild Type - KO mixtures (a-d) Violin plot of the probability score of the top predicted positions by *m6Anet* across the 5 mixtures with different threshold on the minimum probability of modification on the Wild Type samples and KO samples (n=34, 104, 223, and 1042 respectively). The plots still show the expected decrease in the predicted m6A probability as the percentage of METTL3-KO reads increase even with less stringent thresholds. (e) Box plots comparing the ratio of the predicted modification stoichiometry between the HEK293T cell line with different levels of KO mixtures (n=1042) (f-h) HEK293T KO mixtures on transcripts localised to (f) Nucleus (n=79) (g) Ribosome (n=434) (h) Cytosol (n=647). The horizontal lines on the boxes show minima, 25th percentile, median, 75th percentile, and maxima. Points that do not fall within 1.5 times of the interquartile range are considered outliers and are not shown on the plot. The x-axis indicates the percentage of WT reads in the mixture while the vertical lines indicate the expected stoichiometric ratio for each mixture with the matching colour. (i-j) Changes in the concentration of points as visualized on the first two principal components of the same PCA space as in Figure 4. The gradual shifts from (i) to (j) suggests that m6Anet read features capture the expected change in the stoichiometry of m6A modifications (c-d)

1. Koh, C. W. Q., Goh, Y. T. & Goh, W. S. S. Atlas of quantitative single-base-resolution N6-methyl-adenine methylomes. *Nat. Commun.* **10**, 5636 (2019).
2. Linder, B. *et al.* Single-nucleotide-resolution mapping of m6A and m6Am throughout the transcriptome. *Nat. Methods* **12**, 767–772 (2015).
3. Pratanwanich, P. N. *et al.* Identification of differential RNA modifications from nanopore direct RNA sequencing with xPore. *Nat. Biotechnol.* **39**, 1394–1402 (2021).
4. Liu, H. *et al.* Accurate detection of m6A RNA modifications in native RNA sequences. *Nat. Commun.* **10**, 4079 (2019).
