# Supplementary material for: Detection of m6A from direct RNA sequencing using a multiple instance learning framework
Source: Nat Methods. 2022 Nov 10;19(12):1590–8. doi: 10.1038/s41592-022-01666-1 (PMC9718678; doi:10.1038/s41592-022-01666-1)
Supplement: Supplementary file 2 — Reporting Summary [file 41592_2022_1666_MOESM2_ESM.pdf]

nature research

Corresponding author(s): Jonathan Göke, Alexandre Thiery

Last updated by author(s): Sep 27, 2021

Reporting Summary

Nature Research wishes to improve the reproducibility of the work that we publish. This form provides structure for consistency and transparency in reporting. For further information on Nature Research policies, see our Editorial Policies and the Editorial Policy Checklist.

Statistics

For all statistical analyses, confirm that the following items are present in the figure legend, table legend, main text, or Methods section.

n/a	Confirmed	
☐	☒	The exact sample size (*n*) for each experimental group/condition, given as a discrete number and unit of measurement
☐	☒	A statement on whether measurements were taken from distinct samples or whether the same sample was measured repeatedly
☐	☒	The statistical test(s) used AND whether they are one- or two-sided<br>*Only common tests should be described solely by name; describe more complex techniques in the Methods section.*
☒	☐	A description of all covariates tested
☐	☒	A description of any assumptions or corrections, such as tests of normality and adjustment for multiple comparisons
☐	☒	A full description of the statistical parameters including central tendency (e.g. means) or other basic estimates (e.g. regression coefficient) AND variation (e.g. standard deviation) or associated estimates of uncertainty (e.g. confidence intervals)
☐	☒	For null hypothesis testing, the test statistic (e.g. *F*, *t*, *r*) with confidence intervals, effect sizes, degrees of freedom and *P* value noted<br>*Give P values as exact values whenever suitable.*
☒	☐	For Bayesian analysis, information on the choice of priors and Markov chain Monte Carlo settings
☒	☐	For hierarchical and complex designs, identification of the appropriate level for tests and full reporting of outcomes
☒	☐	Estimates of effect sizes (e.g. Cohen's *d*, Pearson's *r*), indicating how they were calculated

Our web collection on statistics for biologists contains articles on many of the points above.

Software and code

Policy information about availability of computer code

Data collection	No software is used for data collection
Data analysis	pandas 1.2.5, numpy 1.20.3, matplotlib 3.3.4, seaborn 0.11.1, pytorch 1.8.1 +cu102, statsmodels 0.12.2, Tombo 1.5.1, EpiNano 1.1, EpiNano 1.2, MINES (https://github.com/YeoLab/MINES), MINES modified (https://github.com/chrishendra93/MINES.git), nanom6A (https://github.com/gaoyubang/nanom6A) , m6anet 0.0.2,m6anet 1.1.0, tensorflow 2.8.0, upsetplot 0.6.0, scikit-learn 1.0.2

For manuscripts utilizing custom algorithms or software that are central to the research but not yet described in published literature, software must be made available to editors and reviewers. We strongly encourage code deposition in a community repository (e.g. GitHub). See the Nature Research guidelines for submitting code & software for further information.

Data

Policy information about availability of data

All manuscripts must include a data availability statement. This statement should provide the following information, where applicable:
- Accession codes, unique identifiers, or web links for publicly available datasets
- A list of figures that have associated raw data
- A description of any restrictions on data availability

The HCT116 cell lines data were obtained through ENA (PRJEB44348) while the HEK293T cell lines data along with its KO variants and KO mixture variants were through ENA (PRJEB40872). The Arabidopsis Virilizer-1 complemented mutant is available through ENA (PRJEB32782) while the curlcakes dataset is available at the GEO database (GSE124309).

April 2020

Field-specific reporting

Please select the one below that is the best fit for your research. If you are not sure, read the appropriate sections before making your selection.

☒ Life sciences ☐ Behavioural & social sciences ☐ Ecological, evolutionary & environmental sciences

For a reference copy of the document with all sections, see nature.com/documents/nr-reporting-summary-flat.pdf

Life sciences study design

All studies must disclose on these points even when the disclosure is negative.

Sample size	We trained and test our model on a total of 10 replicates from HCT116 cell line, HEK293T cell line(1 replicate), HEK293T(1 replicate) with METTL3-KO mixture cell lines (4 cell lines in total) and Arabidopsis VIRC mutants (4 replicates). No sample size calculation was performed, all samples used here have been published before and are publicly available. Each sample contains roughly a million reads,, covering a total of two species which we feel is sufficient to demonstrate the generalisability of our approach
Data exclusions	Data exclusions involve filtering for positions that have less than 20 reads being expressed. This is to reduce prediction noise that can arise from making predictions on sites very few reads
Replication	We have set fixed random seed during training and testing of the data. Code is also available online to reproduce our experiments
Randomization	For each cell line involved in the study, we randomly form training, validation, and testing set, ensuring that each set contains different genes so as to ensure generalizability of the study. Model is then trained on the training set while validation set is used for model selection and test set is used to compare the performance of the models used in this study.
Blinding	Blinding is not relevant to this study as training set and test sets are randomly sampled and we also perform validation on all samples involved by comparing the performance of each model with comparable model trained on the test cell line, thereby ensuring impartiality in our experiments

Reporting for specific materials, systems and methods

We require information from authors about some types of materials, experimental systems and methods used in many studies. Here, indicate whether each material, system or method listed is relevant to your study. If you are not sure if a list item applies to your research, read the appropriate section before selecting a response.

Materials & experimental systems

n/a	Involved in the study
☒	☐ Antibodies
☒	☐ Eukaryotic cell lines
☒	☐ Palaeontology and archaeology
☒	☐ Animals and other organisms
☒	☐ Human research participants
☒	☐ Clinical data
☒	☐ Dual use research of concern

Methods

n/a	Involved in the study
☒	☐ ChIP-seq
☒	☐ Flow cytometry
☒	☐ MRI-based neuroimaging
